# Functional and Bioinformatic Analysis of *PDX2* from *Ginkgo biloba*

**DOI:** 10.3390/genes16050609

**Published:** 2025-05-21

**Authors:** Yifan Xiao, Zhi Feng, Zhi Yao, Hailan Jiang, Yiqiang Wang, Meng Li

**Affiliations:** 1 Key Laboratory of Forestry Biotechnology of Hunan Province, Central South University of Forestry and Technology, Changsha 410004, China; 18975415323@163.com (Y.X.); biotechnologyfeng@163.com (Z.F.); yaosensei@163.com (Z.Y.); 16670498675@163.com (H.J.); 2 Yuelushan Laboratory Carbon Sinks Forests Variety Innovation Center, Changsha 410012, China

**Keywords:** *Ginkgo biloba* L., gene cloning, expression analysis, vitamin B6

## Abstract

**Background**: The *PDX2* gene serves as a critical catalytic component in vitamin B6 (VB6) biosynthesis pathways and plays pivotal regulatory roles in plant growth. **Methods**: To investigate the metabolic regulation of *PDX2* (*GbPDX2*) from *Ginkgo biloba* in VB6 biosynthesis during kernel development, we successfully cloned this gene and conducted systematic expression profiling through qRT-PCR across multiple tissues and developmental stages. **Results**: Bioinformatic characterization revealed that *GbPDX2* contains a 765-bp coding sequence encoding a 254-amino acid polypeptide. The encoded protein displays typical hydrophilic properties (average hydrophobicity index: −0.32) and was predicted to be an unstable cytosolic protein (instability index: 45.7) lacking signal peptides or transmembrane domains with cytoplasmic localization. Phylogenetic analysis demonstrated that *GbPDX2*’s closest evolutionary relationship was with its ortholog in *Picea sitchensis*, which had an amino acid sequence similarity of 83.7% with spruce *PsPDX2*. Tissue-specific expression analysis revealed a gradient expression profile of Kernel > Exocarp > Leaves > Stems > Roots. The expression level in kernels was significantly higher than that in other tissues (19.7 times that in roots, 8.3 times that in stems, and 5.9 times that in leaves; *p* < 0.01), with peak transcript levels observed in mature kernels. HPLC quantification established a strong positive correlation between *GbPDX2* expression dynamics and VB6 accumulation patterns during kernel maturation (r = 0.92, *p* < 0.01), and the peak period of VB6 reached 288.9 ± 7.1 μg/g. **Conclusions**: Our findings provide the first experimental evidence that *GbPDX2* spatiotemporally regulates VB6 biosynthesis in ginkgo kernels, offering novel insights into the evolutionary adaptation of vitamin metabolism in gymnosperms.

## 1. Introduction

Vitamin B6 (VB6), an essential water-soluble vitamin for all organisms [1], serves as a pivotal coenzyme precursor involved in fundamental metabolic processes in plants [2], including amino acid metabolism [3], reactive oxygen species (ROS) scavenging [4], and the biosynthesis of secondary metabolites. Its content directly influences plant stress resistance and medicinal value [4]. The synthesis of vitamin B6 occurs in all parts of plants. Plants can de novo *biosynthesize* vitamin B6 through a deoxyxylose-5-phosphate (DXP)-independent pathway, involving two cytoplasmic enzymes [5], *PDX1* (pyridoxal 5-phosphate synthase activity) and *PDX2* (glutaminase activity) [6,7]. In the initial step, glutamine (Gln) is converted into glutamic acid (Glu) by releasing an active NH_3_ group through the glutaminase activity of the *PDX2* protein. This active NH_3_ group is further activated by the activity of pyridoxal-5 phosphate synthase (*PDX1*) in the cell. It is used for the synthesis of pyridoxal 5-phosphate (PLP) with the help of glyceraldehyde 3-phosphate (G-3-P) and ribose-5-phosphate (R5P) [7,8]. Molecular genetic studies have demonstrated that the *PDX2* gene plays a pivotal regulatory role in the embryonic development of the model plant *Arabidopsis thaliana.* Of particular significance [9], the homozygous mutation of *PDX2* leads to embryonic lethality, underscoring the gene’s indispensable function in plant growth and development. Given the substantial technical challenges in obtaining functional *PDX2* mutants, current research efforts have predominantly focused on the functional characterization of *PDX1* homologs [3,10], with systematic investigations being conducted across multiple laboratories [11,12,13].

*Ginkgo biloba* L., one of the most ancient relict plant species and the sole surviving member of the Ginkgoaceae family, represents a unique model for studying evolutionary adaptations in plant metabolism. This “living fossil” species exhibits extraordinary environmental resilience, attributed to its sophisticated antioxidant systems (total flavonoid content >25 mg/g DW) and unique secondary metabolites. Notably, ginkgo kernels accumulate remarkably high vitamin B6 levels (288.9 μg/g fresh weight), approximately 3-5 times greater than most angiosperm species, and accumulates significantly higher VB6 levels in its kernel than in other tissues [14]. This exceptional accumulation suggests the evolutionary specialization of VB6 metabolism in ginkgo, and the underlying molecular mechanisms remain completely unexplored. Ginkgo also has pharmacologically active flavonoids and terpenoid lactones that exhibit neuroprotective and cardioprotective effects. However, compared with model plants, the molecular regulatory mechanisms underlying VB6 biosynthesis in ginkgo remain unknown, particularly the cloning and functional characterization of key biosynthetic genes [5].

Based on the key role of the *PDX2* gene in the synthesis of vitamin B6 and the characteristic that ginkgo is rich in vitamin B6, we speculate there are functionally conserved *PDX2* homologous genes in ginkgo kernels; *GbPDX2* shows a high expression pattern in its kernels. *GbPDX2* may enhance abiotic stress tolerance in *Ginkgo biloba* by modulating VB6-dependent ROS scavenging pathways given VB6’s known role in oxidative stress mitigation, and the expression level is positively correlated with the content of vitamin B6 in the kernels. Although significant progress has been made in elucidating the regulatory networks of flavonoid and terpenoid biosynthesis in ginkgo [10], the systematic screening and functional analysis of VB6-related genes are still lacking, which severely hinders genetic improvements in medicinal quality and the development of value-added products. In this study, we first cloned the full-length *GbPDX2* gene from ginkgo and characterized its conserved domains and phylogenetic evolution. Using qRT-PCR, we analyzed the expression patterns of *GbPDX2* during seed development, coupled with the HPLC quantification of VB6 accumulation. Our findings not only provide direct evidence for the regulatory role of *GbPDX2* in VB6 biosynthesis but also offer new insights into the evolution of vitamin metabolism in gymnosperms.

## 2. Materials and Methods

### 2.1. Test Materials

The experimental samples were collected from the *Ginkgo biloba “Foshou”* variety (germplasm resource number ZNL-2) cultivated in the Botanical Garden of Central South University of Forestry and Technology (geographical coordinates: 28.13° N, 112.98° E).

### 2.2. Cloning of the GbPDX2 Gene

The RNA of *Ginkgo biloba* was extracted using an RNA extraction kit(E.Z.N.A.TM Plant RNA Kit, Omega Bio-Tek Company, Norcross, GA, USA), and then the integrity was detected by agarose gel electrophoresis with a mass fraction of 1%. The first strand of cDNA was synthesized using a cDNA reverse transcription kit, with reference to the multi-scale integrated ginkgo germplasm database (https://ginkgo.zju.edu.cn/genome/, accessed on 25 December 2024) after extraction of the CDS *GbPDX2* gene sequence, using Primer Premier 5.0 design-specific primers (Table 1). PCR amplification was carried out using the cDNA of *Ginkgo biloba* as a template.

The PCR reaction conditions were as follows: pre-denaturation at 95 °C for 3 min; 95 °C for 30 s, 62 °C for 20 s, and 72 °C for 60 s for 35 cycles. The amplification product was extended at 72 °C for 5 min and stored at 4 °C. The resulting amplification product was subjected to electrophoresis detection using 1% agarose gel (electrophoresis conditions: voltage 120 V, current 220 mA, electrophoresis time 20 min). After detection, it was observed whether the target band was clear and single. The target DNA fragments after the PCR amplification products were detected by agarose gel electrophoresis were retrieved using a column DNA gel recovery kit (Omega Bio-Tek Company, USA). The recovered part remained connected to the vector and was transformed into DH5α *Escherichia coli* for positive screening, with three technical repetitions. After culturing the monoclonal positive bacteria, PCR detection was performed on them. The positive bacterial liquid was selected and transported to the biotechnology laboratory for sequencing. For sequencing verification, bidirectional Sanger sequencing was performed on three independently transformed positive clones, and sequence alignment using DNAMAN achieved 100% consistency. The conserved domain was confirmed through the NCBI CDD database.

### 2.3. Real-Time Fluorescence Quantitative PCR (RT-qPCR)

Using *GAPDH* as the internal reference gene, specific primers for real-time fluorescence quantitative PCR were designed (Table 1). The reaction system was configured according to the instructions of 2×Universal Blue SYBR Green qPCR Master Mix. The amplification procedure was as follows: pre-denaturation at 95 °C for 30 s; and, in phase two, 95 °C for 15 s and 60 °C for 30 s for 40 cycles. After amplification was completed, the temperature increased from 60 °C to 95 °C at a rate of 0.5 °C/10 s, and the fluorescence intensity of the samples was continuously measured to obtain the melting curve. Data analysis was conducted using the 2^−∆∆Ct^ method. All qRT-PCR experiments were set up with three independent biological replicates (samples from different plants) and three technical replicates. One-way analysis of variance (ANOVA) was used to test the significance of differences, and the significance level was set as *p* < 0.05. All significant differences were clearly marked in the Section 3. The melting curve showed a single peak, confirming the absence of primer dimer and non-specific amplification.

### 2.4. The Content of Vitamin B6 in Kernels at Different Developmental Stages

Samples of kernels at different developmental stages were taken, rapidly frozen with liquid nitrogen, and then crushed into homogeneous powder in a grinder. Three biological replicates were set up for each developmental stage. Next, 0.1 g of powder samples were precisely weighed, added to 2 mL pre-cooled centrifuge tubes, and stored in an ultra-low temperature refrigerator at −80 °C for no more than 72 h before analysis. The total content of vitamin B6 was detected using the BC2114 kit (item number BC2114) (Beijing Soleibao Technology Co., Ltd., Beijing, China), strictly following the operational instructions. The peak area of the reaction solution was measured using a high-performance liquid chromatograph, and, finally, the total content of vitamin B6 was calculated.

### 2.5. Bioinformatics Analysis Method of the GbPDX2 Gene

The analysis of genetic bioinformatics was conducted according to the websites or software listed in Table 2.

## 3. Results

### 3.1. Cloning of the GbPDX2 Gene

**Figure 1:**
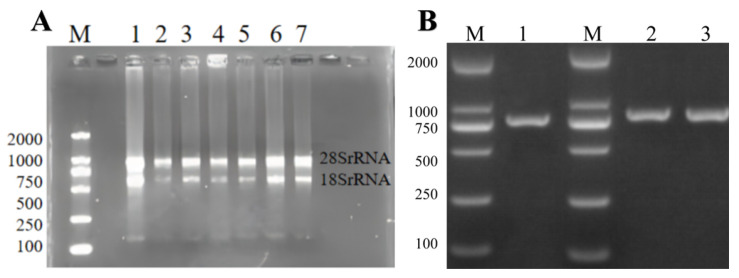
Analysis of RNA extraction and *GbPDX2* gene cloning results: (**A**) lanes 1~7 are all RNA, M: DL2000 DNA marker; (**B**) PCR amplification results for lane 1; PCR amplification results of *Escherichia coli* colonies in lanes 2~3, M: DL2000 DNA marker.

The total RNA of ginkgo was extracted using the E.Z.N.A.TM Plant RNA kit (Omega Bio-Tek Company, USA). The results of agarose gel electrophoresis showed that in Figure 1, both the 28S rRNA band and the 18S rRNA band were clearly visible in the electrophoresis results map of the total RNA of the sample. This indicated that the extracted total RNA was relatively complete. The OD260/OD280 value of the extracted RNA was between 1.9 and 2.0, indicating that the RNA purity was relatively high. This could be used for subsequent experiments.

Using the cDNA of ginkgo kernels as a template, PCR amplification was performed with specific primers. The amplification products were detected by 1% agarose gel to obtain a clear and single target fragment, with a fragment size of 765bp. After gel recovery and purification, a clone vector was constructed to transform DH5*α Escherichia coli*. A single positive clone plaque was screened. After expanding the culture, the plasmid was extracted for sequencing. Finally, the target gene sequence was cloned. After sequencing, it was consistent with the target fragment, indicating the success of gene cloning. The cloned sequence was named *GbPDX2.*

### 3.2. Sequence Analysis of GbPDX2 Proteins

**Figure 2:**
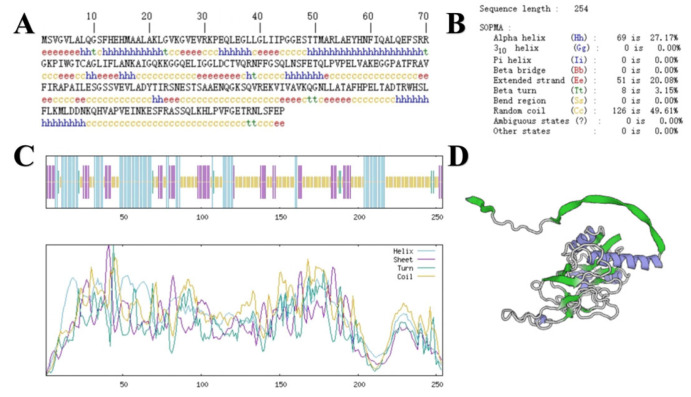
Prediction of the structure of the *GbPDX2* protein: (**A**) primary structure of the *GbPDX2* protein; (**B**,**C**) prediction of the secondary structure of the *GbPDX2* protein; (**D**) prediction of the tertiary structure of the *GbPDX2* protein.

The sequence size of the *GbPDX2* gene was 765bp, encoding 254 amino acids. The primary structure of its protein is shown in Figure 2. ExPASy was used to predict the basic physicochemical properties of the *GbPDX2* protein. The results show that the molecular formula of this protein was C_1236_H_1971_N_345_O_363_S_6_, the molecular weight was 27,664.70 Da, the theoretical isoelectric point (PI) was 7.82, the total number of atoms was 3921, and the amino acid with the highest proportion was leucine (Leu), accounting for 11%, while cysteine (Cys) had the lowest content, accounting for only 0.8%. The total number of negatively charged amino acid residues (Asp + Glu) was 26, the total number of positively charged amino acid residues (Arg + Lys) was 27, the fat coefficient was 91.42, and the instability index was 45.86, making it an unstable protein.

The secondary structure of the *GbPDX2* protein of ginkgo was predicted using SOPMA. Among the 254 amino acids of the *GbPDX2* protein, the irregular curl was composed of 126 amino acids, accounting for 49.61%. The α-helix was formed of 69 amino acids, accounting for 27.17%. Fifty-one amino acids were extended chains, accounting for 20.08%. Eight amino acids were β -turn, accounting for 3.15%.

The tertiary structure of the *GbPDX2* protein was predicted by SWISS-MODEL and visualized by Pymol software. The results are shown in Figure 2. The *GbPDX2* protein is mainly an α-helix with irregular coiling, which is consistent with the prediction results of the secondary structure.

### 3.3. Characterization of GbPDX2 Proteins

**Figure 3:**
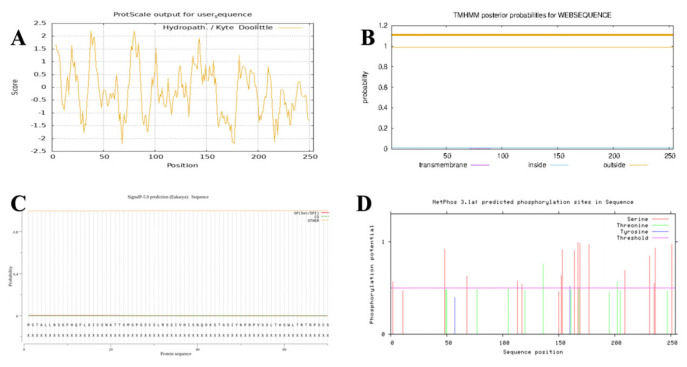
The characterization of *GbPDX2* proteins: (**A**) hydrophilic/hydrophobic analysis; (**B**) prediction of the transmembrane domain; (**C**) signal peptide prediction; (**D**) prediction of the phosphorylation site.

The hydrophilicity and hydrophobicity of the *GbPDX2* protein were predicted using ProtScale (Figure 3). According to the principle that the hydrophilic peak is less than 0 and the hydrophobic peak is greater than 0, the results indicated that glycine (Gly), at the 80th position of the polypeptide chain, had the strongest hydrophobicity with a score of 2.211, and serine (Ser), at the 68th position, had the strongest hydrophilicity. The scores were all −2.211. There were more hydrophilic amino acids in the entire peptide chain than hydrophobic amino acids. Moreover, ProtParam analysis indicated that the total average hydrophobic index (GRAVY) was −0.130. It was speculated that the *GbPDX2* protein was a hydrophilic protein. The transmembrane domain of the *GbPDX2* protein was predicted using TMHMM2.0. The results showed that the entire peptide chain did not have a transmembrane domain, so it was not a membrane protein.

The signal peptide sites of the *GbPDX2* protein were analyzed using signal5.0. The results showed that there were no obvious peaks in the original splicing site score (C-score) or the comprehensive splicing site score (Y-score) between the N-terminal and the 70th amino acid. In this figure, the orange line remains below 0.1. This means that SignalP-5.0 predicts that this protein sequence has no signal peptide region. In other words, this protein may not be a secretory protein, or its signal peptide is so short or atypical that Signal5.0 cannot detect it.

Protein phosphorylation is one of the important covalent modification processes commonly present in living organisms and is closely related to signal transduction and growth and development. The phosphorylation sites of the *GbPDX2* protein were predicted using the Netphos 3.1 server. The results showed that there were 32 sites where phosphorylation might occur in the amino acid sequence encoded by the *GbPDX2* gene of Ginkgo biloba. There were 18 sites where serine (Ser) may undergo phosphorylation, located at positions 2, 11, 48, 68, 113, 117, 150, 152, 153, 164, 167, 169, 177, 209, 231, 235, 236, and 251 of the amino acid sequence. There were 12 sites where threonine (Thr) may undergo phosphorylation, located at positions 49, 50, 77, 105, 120, 136, 161, 168, 195, 202, 205, and 247 of the amino acid sequence. There were two sites where tyrosine (Tyr) may undergo phosphorylation, located at the 57th and 160th amino acids in the amino acid sequence.

### 3.4. Prediction of Conserved Domains, Cluster Analysis, and the Multiple Sequence Alignment of the GbPDX2 Protein

**Figure 4:**
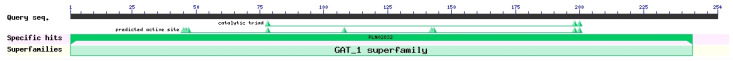
Prediction of the conserved domain of the *GbPDX2* protein.

The conserved domain prediction of the *GbPDX2* protein sequence was performed using CDD (Conserved Domain Database) in NCBI (Figure 4). The results showed that *GbPDX2* was predicted to belong to the GAT-1 superfamily and was annotated as type 1 glutamintransferase. The 1-244aa of the *GbPDX2* protein sequence contained PLN02832 (glutamine amidotransferase subunit of pyridoxal 5′-phosphate synthase complex). Pyridoxal-5’-phosphate synthase complex glutamintransferase subunit conserved domains are members of the trivalent glutamintransferase family and play an important role in the biosynthetic pathway of vitamin B6.

**Figure 5:**
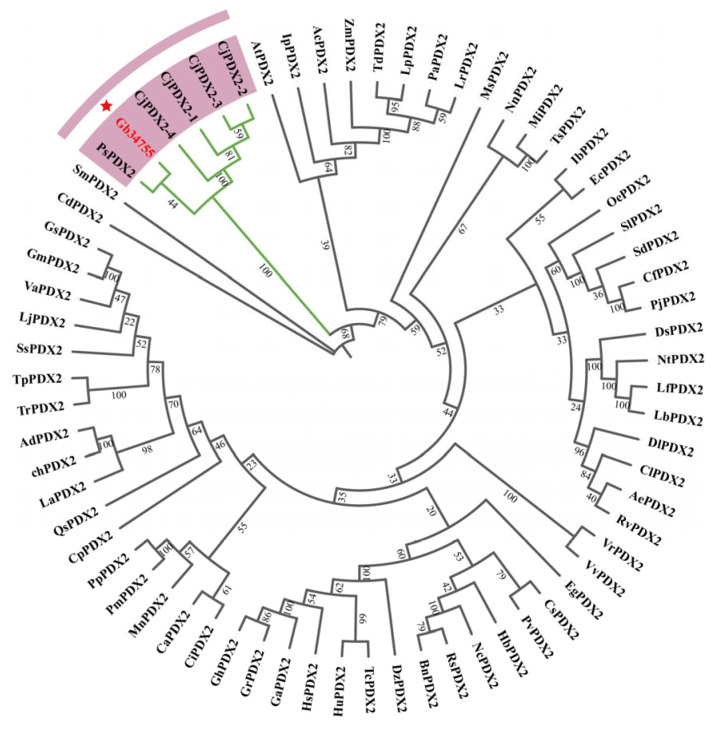
Phylogenetic analysis of *GbPDX2* and *PDX2* genes in other species.

In order to study the evolutionary relationship between ginkgo *PDX2* and the model species *Arabidopsis thaliana* and other species, the sequences with high homology to *GbPDX2* were analyzed by BLAST in the NCBI protein database and downloaded. The evolutionary tree was constructed by the adjacency method using MEGA-X software, and the evolutionary tree was visualized using ChipLot online (Figure 5). It can be seen from the cluster analysis diagram that the *GbPDX2* protein of the ginkgo gymnosperm is clustered into one branch together with the *PsPDX2* protein of *Picea sitchensis* and the *CjPDX2* protein of *Cryptomeria japonica*, and they are closely related. Among them, it has the closest genetic relationship with the *PsPDX2* protein of spruce.

**Figure 6:**
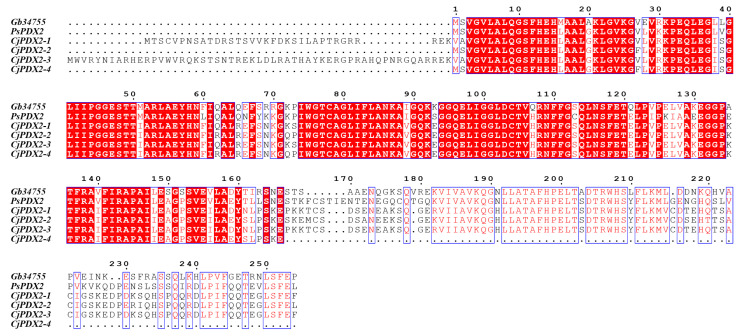
Multiple amino acid sequence comparison between GbPDX2 and PDX2 of other species.

The amino acid sequences of the *GbPDX2* protein of ginkgo were compared with those of the *PDX2* amino acid sequences of closely related species, showing a homology of 71.26–83.73%. Multi-sequence alignment was performed using DNAMAN 5.0 software and plotted with BioEdit, and the alignment results are shown in Figure 6. The results indicate that the N-terminus of their sequences all have the glutamintransferase subunit conserved domain of the pyridoxal-5’-phosphate synthase complex, which is used for the biosynthesis of vitamin B6, demonstrating the conservation of *PDX2* during the evolutionary process.

### 3.5. Prediction and Analysis of cis-Acting Elements in the GbPDX2 Promoter

**Figure 7:**
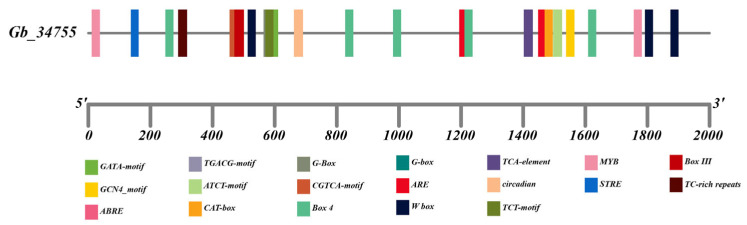
Prediction of the cis-acting elements of the *GbPDX2* promoter.

The promoter region of the *GbPDX2* gene is enriched with diverse cis-acting elements (in Figure 7), which are likely to play critical roles in regulating the gene’s functions related to light responsiveness, hormone signal transduction, stress responses, and tissue-specific expression. Notably, the presence of multiple light-responsive elements suggests that the expression of this gene may be substantially modulated by varying light conditions. These observations offer valuable insights and lay a solid foundation for further investigation into the transcriptional regulatory mechanisms underlying *GbPDX2* gene expression.

### 3.6. The Expression Pattern of the GbPDX2 Gene in Different Tissues and at Different Developmental Stages of Ginkgo biloba

**Figure 8:**
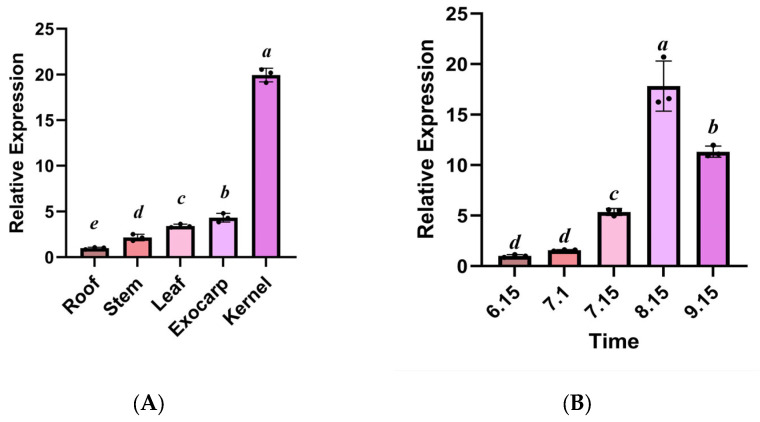
Analysis of the expression pattern of *GbPDX2*: (**A**) analysis of *GbPDX2* gene expression in different tissues; (**B**) analysis of expression levels of ginkgo kernels at different developmental stages. Bars are means ± SD of three independent experiments, with different letters indicating a statistically significant difference; *p* < 0.05.

Quantitative analysis by qRT-PCR revealed that the *GbPDX2* gene of *Ginkgo biloba* showed a significant tissue-specific expression preference (*p* < 0.05). In the reproductive organs, the transcriptional abundances of the kernel and exocarp were 19.70 times and 4.48 times those of the root tissue, respectively, and the difference between the two reached a statistically significant level (in Figure 8). It is worth noting that the peak expression of *GbPDX2* during the kernel maturity period (15 August) was synchronized with the key stage of VB6 synthesis, suggesting that it may regulate the redirection of kernel metabolic flux through a dose effect. Furthermore, the gene in vegetative organs showed a hierarchical expression characteristic (Leaf > Stem > Root), in which the expression level in leaves is 3.32 times that in roots, suggesting that it may be involved in the synthesis–transport coupling mechanism of leaf-derived vitamins. The expression level of the *PDX2* gene showed a nonlinear growth pattern during kernel development. It began to rise slowly from 15 June, reached an expression peak on 15 August, which was significantly higher (17.8 times) than the initial level (*p* < 0.001), and then slightly decreased on 15 September. From 15 July to 15 August, the expression level of *PDX2* showed a sharp upward trend (increasing by 3.3 times), and this explosive growth pattern was highly consistent with the rapid development stage of ginkgo seeds. This expression characteristic suggests that *PDX2* may act as a key regulatory factor to participate in the adaptation of vitamin B6 during kernel maturation, especially playing an important role during the critical period of accumulation of storage substances.

### 3.7. Determination of the Total Vitamin B6 Content in Ginkgo Kernels at Different Developmental Stages

**Figure 9:**
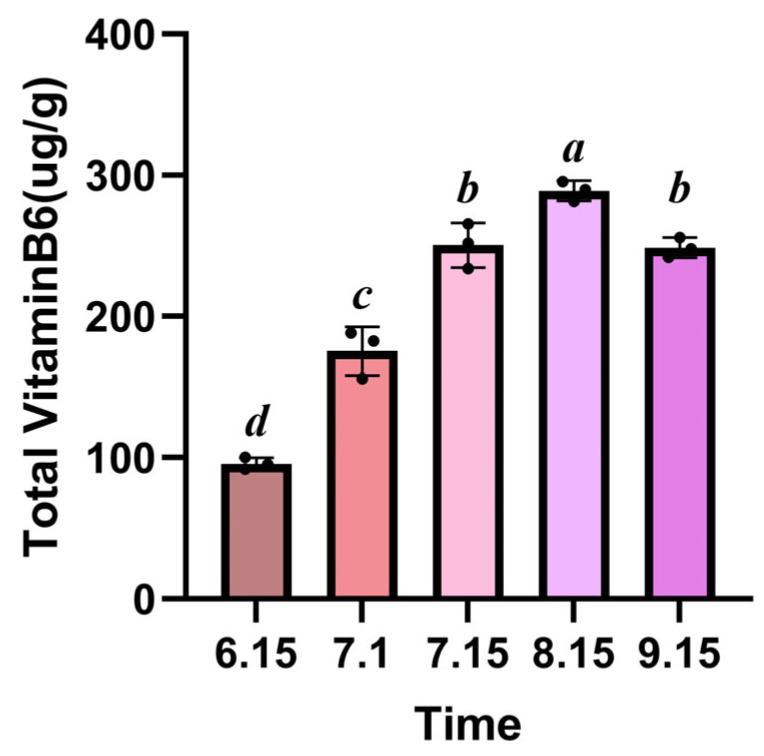
Content of vitamin B6 in ginkgo kernels at different developmental stages. Bars are means ± SD of three independent experiments, with different letters indicating statistically significant differences; *p <* 0.05.

HPLC quantitative analysis showed that the content of vitamin B6 in ginkgo kernels presented dynamic changes during development (Figure 9). The content of VB6 gradually increased from the early stage of development (6.15:95.7 ± 4.3 μg/g), reaching a peak at 8.15 (288.9 ± 7.1 μg/g), which was significantly higher (3.02 times) than at 6.15 (*p* < 0.01). It is worth noting that the content of VB6 at 9.15 (248.7 ± 7.1 μg/g) was slightly decreased compared with the peak but still remained at a relatively high level, indicating that the accumulation process of VB6 showed a trend of first increasing and then slightly decreasing. This dynamic change pattern is highly consistent with the critical period of storage substance accumulation during the development of ginkgo kernels, suggesting that VB6 may act as an important coenzyme to participate in metabolic regulation during the kernel maturation process. This result provides direct evidence for analyzing the temporal nature of VB6 synthesis in ginkgo kernels.

## 4. Discussion

This study provides the first comprehensive molecular characterization of *GbPDX2* in *Ginkgo biloba*, elucidating its spatiotemporal regulatory role in vitamin B6 (VB6) biosynthesis during kernel development. Our findings not only expand the understanding of VB6 metabolic regulation in gymnosperms but also offer critical insights into the potential adaptive significance of *PDX2* in plant stress biology.

Vitamin B6, an endogenous growth regulator in plants, functions as an antioxidant that modulates physiological metabolism, growth, developmental processes, and stress responses. The *PDX* gene family plays a pivotal role in the vitamin B6 biosynthesis pathway and has been demonstrated to mediate plant adaptation to diverse environmental stresses. For instance, in *Arabidopsis thaliana*, *PDX1* and *PDX2* are upregulated under strong light, cold, drought, and ozone stress, whereas the *PDX1.3* mutant exhibits heightened sensitivity to salt, oxidative, and osmotic stresses.

Current research on plant *PDX* genes has primarily focused on *Arabidopsis thaliana*. However, emerging evidence suggests that *GbPDX2* in *Ginkgo biloba* may contribute to abiotic stress tolerance beyond its canonical role in vitamin B6 synthesis. Vitamin B6 (e.g., pyridoxal-5′-phosphate, PLP) acts as a potent antioxidant, scavenging reactive oxygen species (ROS) generated under drought, salinity, or UV radiation. The elevated expression of *GbPDX2* in metabolically active tissues—such as during seed maturation and photosynthesis—aligns with the increased demand for ROS detoxification. Notably, the high instability index of *GbPDX2* (45.7) suggests rapid protein turnover, which could facilitate dynamic oxidative stress regulation.

In *Arabidopsis*, impaired ROS homeostasis in *PDX2* mutants leads to hypersensitivity to osmotic stress [15,16], whereas *PDX1* overexpression enhances stress tolerance. Given the conserved glutaminase activity of *GbPDX2*, we propose that it sustains *Ginkgo biloba*’s stress resilience by maintaining PLP-dependent enzyme activities (e.g., alanine aminotransferase), which are critical for nitrogen metabolism and antioxidant synthesis. Future studies should validate this hypothesis by profiling *GbPDX2* expression under stress conditions and correlating it with ROS levels and vitamin B6 intermediate dynamics.

Bioinformatic analysis revealed that *GbPDX2* encodes a hydrophilic, cytosolic protein lacking transmembrane domains and signal peptides, consistent with its predicted role in cytoplasmic VB6 biosynthesis [17]. The high sequence homology (71.26–83.73%) and phylogenetic clustering with *Picea sitchensis* and *Cryptomeria japonica* orthologs suggest the strong evolutionary conservation of *PDX2* function in gymnosperms. This conservation may reflect its indispensable role in core metabolic processes, particularly in nitrogen assimilation and VB6-dependent enzymatic reactions [5]. Notably, the instability index (45.7) of *GbPDX2* implies rapid turnover, potentially facilitating dynamic regulation during developmental transitions or stress responses [12].

The tissue-specific expression gradient (Kernel > Exocarp > Leaves > Stems > Roots) aligns with the elevated VB6 accumulation in kernels, supporting *GbPDX2* as a key driver of VB6 biosynthesis in kernels. The sharp transcriptional upregulation during kernel maturation (peaking on 15 August) coincides with the critical phase of storage compound accumulation, suggesting *GbPDX2* mediates metabolic flux toward VB6 production to meet coenzyme demands for seed development [7]. The subsequent decline in VB6 content post-maturity may reflect its conversion into pyridoxal phosphate (PLP), a ROS scavenger critical for seed dormancy [1]. These dynamics align with VB6′s established role as a “metabolic tuner” in angiosperms [7], yet ginkgo’s exceptional kernel VB6 levels (288.9 μg/g) suggest gymnosperm-specific adaptations.

Beyond its metabolic role, VB6 is a well-documented stress protectant, functioning primarily through PLP-dependent enzymes involved in reactive oxygen species (ROS) detoxification [2]. The high *GbPDX2* expression in the kernel and exocarp—tissues prone to oxidative stress during development—hints at its potential role in mitigating abiotic stress. VB6 derivatives, such as pyridoxine, directly scavenge ROS [3], while PLP-dependent enzymes (e.g., alanine aminotransferase) modulate amino acid metabolism under stress [11]. The presence of light-responsive cis-elements in the *GbPDX2* promoter further suggests its regulation by photooxidative stress, a common non-biotic challenge in perennial plants.

While this study establishes *GbPDX2* as a regulator of VB6 biosynthesis, several questions remain unresolved: Does *GbPDX2* overexpression enhance ROS scavenging capacity? (Isotope labeling and mutant analyses are needed to trace VB6 flux under stress.) How do hormonal or environmental cues (e.g., light, drought) modulate *GbPDX2* expression in kernels versus leaves? Does the high VB6 investment in ginkgo kernels compromise stress resilience in vegetative tissues? Comparative studies across gymnosperms could clarify this.

## 5. Conclusions

While this study provides novel insights into the functional characterization of *GbPDX2* in *Ginkgo biloba*, several limitations should be noted. First, the precise regulatory mechanisms governing the spatiotemporal expression patterns of *GbPDX2*, particularly the functional significance of the predicted cis-acting elements in its promoter region, remain to be fully elucidated. Furthermore, while we identified potential phosphorylation sites, the biological relevance of these post-translational modifications in modulating *GbPDX2* activity and VB6 biosynthesis requires experimental validation. The current lack of established genetic transformation protocols for *Ginkgo biloba* presents a significant technical barrier to conducting functional validation through gene knockout or overexpression studies.

Future investigations should prioritize the following: (1) the identification and characterization of transcription factors that regulate *GbPDX2* expression in response to environmental stimuli; (2) comprehensive analysis of VB6 metabolic flux during seed dormancy and germination using stable isotope labeling approaches; and (3) the development of genetic manipulation tools to enable functional genomic studies in ginkgo. Addressing these knowledge gaps will significantly advance our understanding of the evolutionary conservation and metabolic regulation of VB6 biosynthesis in gymnosperms while providing potential strategies for metabolic engineering to enhance the nutritional value of ginkgo kernels. This study provides the first comprehensive molecular characterization of *GbPDX2* in *Ginkgo biloba*, establishing its critical role in VB6 biosynthesis during kernel development. Our bioinformatic analyses demonstrate that *GbPDX2* encodes a hydrophilic, cytosolic protein with conserved glutaminase activity, consistent with its function in the DXP-independent VB6 pathway. Phylogenetic reconstruction revealed a close evolutionary relationship between *GbPDX2* and its orthologs in other gymnosperms, highlighting the remarkable conservation of VB6 metabolic pathways in this ancient plant lineage.

Expression profiling uncovered a distinct tissue-specific expression gradient (Kernel > Exocarp > Leaves > Stems > Roots) and a developmentally regulated expression pattern, with maximal transcript abundance coinciding with peak VB6 accumulation during kernel maturation. These findings collectively demonstrate that *GbPDX2* serves as a key regulatory node in VB6 biosynthesis in *Ginkgo biloba*, providing important insights into the metabolic specialization of gymnosperms. The strong correlation between *GbPDX2* expression and VB6 content suggests its potential as a target for metabolic engineering approaches aimed at enhancing the nutritional quality of ginkgo kernels.

This work not only advances our understanding of vitamin metabolism in non-model woody plants but also contributes to the broader field of secondary metabolic regulation in gymnosperms. The findings establish a foundation for future studies investigating the evolutionary and functional diversification of vitamin biosynthesis pathways in plants.

## Figures and Tables

**Table 1:** Primer sequence for PCR amplification.

Primer Name	Primer Sequence (5′→3′)
*Gb* *PDX2-F*	ATGTCTGTAGGAGTTTTGGCCCTT
*Gb* *PDX2-R*	TCATGGCTCAAAACTTAAGTTTCTTGTTTC
*q GAPDH-F*	CAAGGACTCCAACACCTTACTC
*q GAPDH-R*	CCGTGGATTCAACCACATACT
*q Gb* *PDX2-F*	TGAAGTAAGGAAACCCGAGCAA
*q Gb* *PDX2-R*	TTGGACTGTACAATCAAGCCCTC

**Table 2:** Bioinformatics analysis tools.

Analysis of Website or Software	Analysis Content
https://web.expasy.org/protparam/ (accessed on 14 February 2025)	physicochemical properties analysis
https://web.expasy.org/protscale/ (accessed on 14 February 2025)	hydrophobicity/hydrophilicity prediction
https://services.healthtech.dtu.dk/services/TMHMM-2.0/ (accessed on 14 February 2025)	transmembrane domain analysis
https://services.healthtech.dtu.dk/services/SignalP-5.0/ (accessed on 14 February 2025)	signal peptide site analysis
https://services.healthtech.dtu.dk/services/NetPhos-3.1/ (accessed on 14 February 2025)	phosphorylation site prediction
https://www.ncbi.nlm.nih.gov/cdd (accessed on 14 February 2025)	conserved domain analysis
https://blast.ncbi.nlm.nih.gov/Blast.cgi (accessed on 14 February 2025)	homology alignment
https://npsa-prabi.ibcp.fr/cgi-bin/npsa_automat.pl?page=npsa_sopma.html (accessed on 14 February 2025)	secondary structure prediction
https://swissmodel.expasy.org/ (accessed on 14 February 2025)	tertiary structure prediction
DNAMAN 5.0	protein sequence alignment
MEGA-X	phylogenetic analysis

## Data Availability

The original contributions presented in this study are included in the article. Further inquiries can be directed to the corresponding authors.
